# Expression and distribution of extensins and AGPs in susceptible and resistant banana cultivars in response to wounding and *Fusarium oxysporum*

**DOI:** 10.1038/srep42400

**Published:** 2017-02-20

**Authors:** Yunli Wu, Wei Fan, Xiaoquan Li, Houbin Chen, Tomáš Takáč, Olga Šamajová, Musana Rwalinda Fabrice, Ling Xie, Juan Ma, Jozef Šamaj, Chunxiang Xu

**Affiliations:** 1 College of Horticulture, South China Agricultural University, Guangzhou 510642, China; 2 Institute of Biotechnology, Guangxi Academy of Agricultural Sciences, Nanning 530007, China; 3 Centre of the Region Haná for Biotechnological and Agricultural Research, Faculty of Science, Palacký University, Šlechtitelů 27, 783 71 Olomouc, Czech Republic

## Abstract

Banana *Fusarium* wilt caused by *Fusarium oxysporum* f. sp. *cubense (Foc*) is soil-borne disease of banana (*Musa* spp.) causing significant economic losses. Extensins and arabinogalactan proteins (AGPs) are cell wall components important for pathogen defence. Their significance for *Foc* resistance in banana was not reported so far. In this study, two banana cultivars differing in *Foc* sensitivity were used to monitor the changes in transcript levels, abundance and distribution of extensins and AGPs after wounding and *Foc* inoculation. Extensins mainly appeared in the root cap and meristematic cells. AGPs recognized by JIM13, JIM8, PN16.4B4 and CCRC-M134 antibodies located in root hairs, xylem and root cap. Individual AGPs and extensins showed specific radial distribution in banana roots. At the transcript level, seven extensins and 23 AGPs were differentially expressed between two banana cultivars before and after treatments. Two extensins and five AGPs responded to the treatments at the protein level. Most extensins and AGPs were up-regulated by wounding and pathogen inoculation of intact plants but down-regulated by pathogen attack of wounded plants. Main components responsible for the resistance of banana were *MaELP*-*2* and *MaPELP*-*2*. Our data revealed that AGPs and extensins represent dynamic cell wall components involved in wounding and *Foc* resistance.

Plant cell walls represent first obstacle that pathogens need to overcome in order to penetrate inside the host cells. Arabinogalactan proteins (AGPs) and extensins are two important members of hydroxyproline-rich glycoprotein (HRGP) superfamily abundant in plant cell walls^1^,^2^. Many recent studies are devoted to biological roles of AGPs in a wide range of plant processes. However, little attention has been given to their role in plant-microbe interactions, particularly in roots^3^. Several studies showed that HRGPs are involved in host-pathogen interactions^2^,^3^,^4^,^5^,^6^,^7^,^8^,^9^,^10^,^11^,^12^ or wounding responses^13^,^14^,^15^. Nevertheless, none of these studies exploited systematic approach to monitor multiple AGPs and extensins by combining data on their transcript levels, abundances and subcellular distribution. Immuno-histochemical techniques using well-defined antibodies are feasible to better define plant cell wall components and to localize precisely cell wall polymers *in situ* within complex tissues^16^. In addition, the development of modern bio-techniques could effectively complement the localisation studies on the single HRGP components.

Banana (*Musa* spp.) is one of the most important fruit and food crops in the world with annual production of more than 100 Mt^17^. *Fusarium* wilt caused by *Fusarium oxysporum* f. sp. *cubense (Foc*) is one of the most destructive diseases substantially reducing the production of banana^18^. Therefore, it is of both biological and agricultural importance to understand the molecular mechanism of banana resistance to *Foc*. In recent years, great efforts revealed molecular mechanism of plant responses to *Foc* by modern bio-techniques, such as transcriptomics, digital gene expression (DGE) and proteomics^19^,^20^,^21^,^22^,^23^. However, to our knowledge, none of these reports studied effects of wounding which was considered as a major factor in this disease infection^24^. Hence, the responses of banana to wounding were considered as very important for the resistance of banana to the pathogen^25^. In *Foc* infected field, most banana roots are usually intact while some might be wounded due to insects or agricultural activities. Therefore, in the present study, we monitor changes in transcript levels, abundances and localization of several extensins and AGPs in two banana cultivars differing in *Foc* susceptibility after wounding and pathogen inoculation, using both intact and wounded plants. Our study revealed that gene expression and subcellular distribution of extensins and AGPs represent important parameters determining *Foc* resistance or susceptibility in banana. Moreover, extensins and AGPs are promising markers which potentially may be used for selecting new resistant varieties and thus accelerate banana breeding efforts.

## Results

### The subcellular distribution of AGPs and extensins in banana roots

#### Extensins

Immunolabelling with five antibodies binding to plant extensins, namely JIM11, JIM12, JIM19, JIM20 and LM1 were used to reveal the subcellular distribution of their antigens in banana roots. Nevertheless only the epitopes of JIM11 and JIM20 were detected in banana roots.

##### Distribution of the epitope of JIM11 antibody

**Figure 1:**
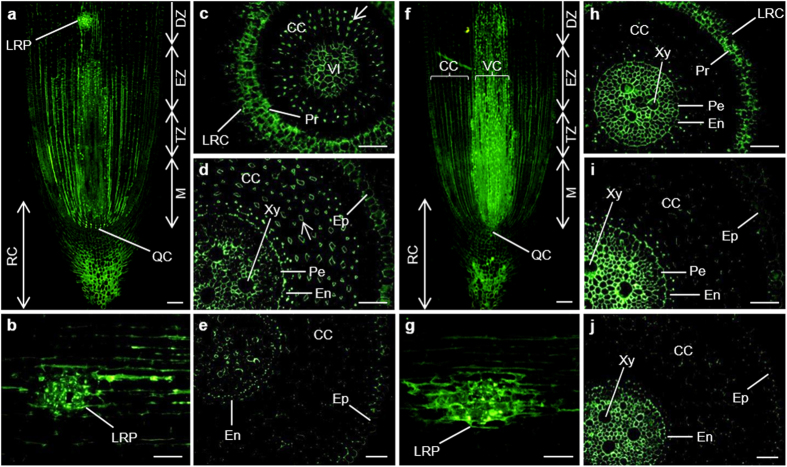
Immunolocalization of JIM11 (**a**–**e**) and JIM20 (**f**–**j**) epitopes in longitudinal and transversal sections of banana (*Musa* spp. AAA) roots. CC, cortical cells; DZ, differentiation zone; En, endodermis; Ep, epidermis; EZ, elongation zone; LRC, lateral root cap; LRP, lateral root primordium; M, meristem; Pe, pericycle; Pr, protoderm; QC, quiescent centre; RC, root cap; TZ, transition zone; VI, vascular initials; VC, vascular cylinder; Xy, xylem. Arrows point to tricellular cell–cell junction. Bars represent 100 μm (**a**,**b**,**f**,**g**) and 50 μm (**c**–**e**,**h**–**j**), respectively.

As shown in Fig. 1, the JIM11 recognizing extensin appeared mainly in the root cap, the meristem, the transition and elongation root zones. Strong signal could be observed in cells where lateral root initiated, the lateral root primordium (Fig. 1a,b). In the cross section of meristem, the signal appeared in cells of the lateral root cap (LRC) and vascular initials (Fig. 1c). In the elongation zone, the signal was present in the epidermis, cortical cells, endodermis and vascular cylinder, with relatively stronger one in the cell–cell junctions of the endodermis. In the cortical cells, the signal was only present in the tricellular cell–cell junctions (an arrow). However, pericycle cells and one or two layers of cortical cells adjacent to the epidermis were not labelled (Fig. 1d). Very weak labelling intensity was observed in differentiation zone and signal nearly disappeared in cortical cells (Fig. 1e).

##### Distribution of the epitope of JIM20 antibody

Immunolocalisation of extensins using JIM20 antibody revealed the strongest signal in the vascular cylinder, followed by the root cap and cortical cells. In the vascular cylinder, the highest antigen level was observed in meristem and transition zone (Fig. 1f). The signal was also observed in the cells of lateral root primordium (Fig. 1g). As shown in the cross sections of meristem, the signal in the LRC, protoderm and central cylinder was strong (Fig. 1h). On the other hand, cortical cells showed nearly undetectable signal. In elongation zone and differentiation zone, the signal in the xylem of the vascular cylinder was the strongest, followed by the endodermis, whereas the signal in cortical cells and epidermis was very weak (Fig. 1i,j). In vascular cylinder, the signal in elongation zone was stronger than in both meristem and differentiation zone (Fig. 1h–j).

#### AGPs

Immunolabelling with 16 antibodies recognizing AGPs were used to reveal the subcellular distribution of AGPs in banana roots. The epitopes of CCRC-M134, LM2, LM14, PN16.4B4, JIM8, JIM13 and JIM16 could be detected in banana roots.

##### The epitopes of LM2 and LM14 antibodies

**Figure 2:**
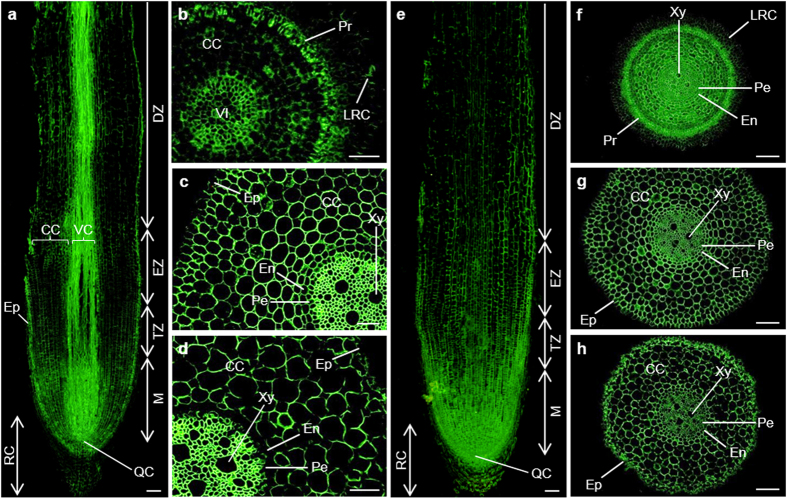
Immunolocalization of LM2 (**a**–**d**) and LM14 (**e**–**h**) epitopes in the cross and longitudinal sections of banana (*Musa* spp. AAA) roots. CC, cortical cells; DZ, differentiation zone; En, endodermis; Ep, epidermis; EZ, elongation zone; LRC, lateral root cap; M, meristem; Pe, pericycle; Pr, protoderm; QC, quiescent centre; RC, root cap; TZ, transition zone; VI, vascular initials; VC, vascular cylinder; Xy, xylem. Bars represent 100 μm (**a**,**e**–**h**) and 50 μm (**b**–**d**), respectively.

In the longitudinal section of banana root, the content of LM2 epitope (β-D-GlcpA) in the meristematic cells and the cells of vascular cylinder was relatively high, while that in the epidermis cells, root cap and cortical cells was much lower (Fig. 2a). In the cross section of meristem, relatively stronger signal could be observed in the protoderm and the vascular initials (Fig. 2b). In the elongation root zone, a very strong signal could be observed in the vascular cylinder, lower one in cortical cells, while that in the epidermis was very weak (Fig. 2c). Similar labelling pattern was observed in differentiation zone, but the signal in the cortical cells of this zone was much weaker, furthermore, no signal could be detectable in the endodermis (Fig. 2d).

As shown in Fig. 2e, the LM14 epitope was present in all root cells in the longitudinal section. The staining intensity of the antigen in meristem and root transition zone was higher than in the root cap, elongation and differentiation zones. As observed in the cross sections from different root zones, the epitope of this antibody was evenly distributed in all sections (Fig. 2f–h) except two cortical cell layers close to the protoderm and LRC in meristem (Fig. 2f), and outer cell walls of epidermis in elongation zone (Fig. 2g).

##### The epitope of JIM16 antibody

**Figure 3:**
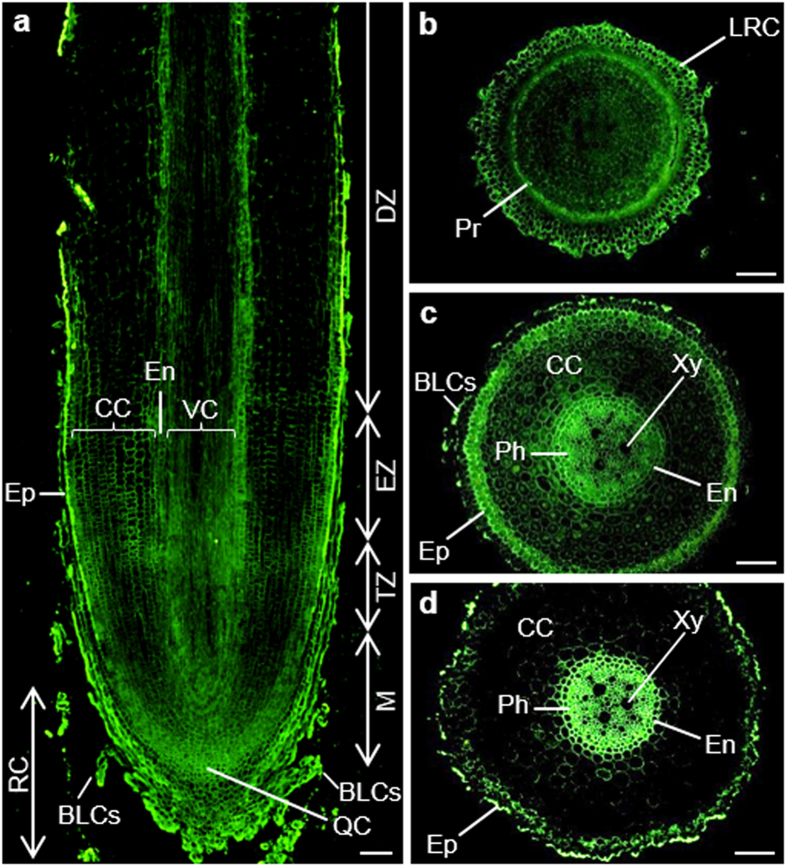
Immunolocalization of JIM16 epitope in the cross and longitudinal sections of banana (*Musa* spp. AAA) roots. BLCs, border-like cells; CC, cortical cells; DZ, differentiation zone; En, endodermis; Ep, epidermis; EZ, elongation zone; LRC, lateral root cap; M, meristem; Ph, phloem; Pr, protoderm; QC, quiescent centre; RC, root cap; TZ, transition zone; VC, vascular cylinder; Xy, xylem. Bars represent 100 μm.

The signal of JIM16 epitope was quite strong in banana roots (Fig. 3). As shown in the longitudinal section (Fig. 3a), very strong signal could be observed in the root border-like cells (BLCs) and epidermis. However, the signal inside was much weaker, with relatively stronger one in the quiescent centre, meristem and cells around the endodermis (Fig. 3a). In the cross section of meristem, the strongest signal was present in the protoderm and LRC (Fig. 3b). In the elongation zone, very strong signal appeared in the BLCs, epidermis, endodermis and the phloem cells. On the other hand, very weak signal was detected in the cortical cells (Fig. 3c). The labelling pattern of differentiation zone was similar to that of elongation zone. However, the signal in differentiation zone was much stronger in outer cell walls of epidermis and in the phloem (Fig. 3d).

##### The epitopes of JIM13, JIM8, PN16.4B4 and CCRC-M134 antibodies

**Figure 4:**
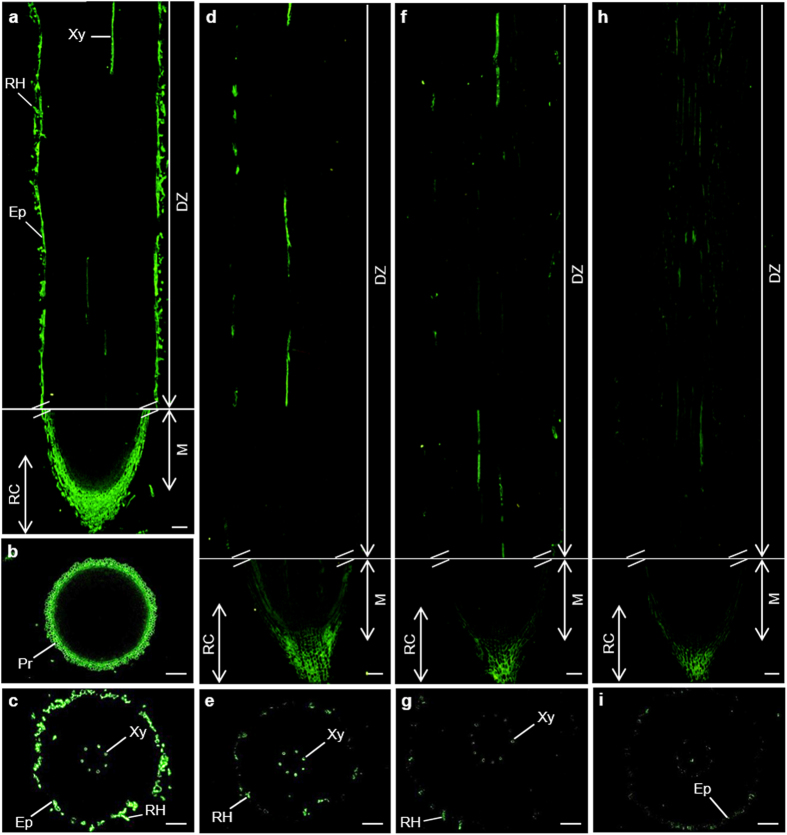
Immunolocalization of JIM13 (**a**–**c**), JIM8 (**d**,**e**), PN16.4B4 (**f**,**g**) and CCRC-M134 (**h**,**i**) epitopes in the cross and longitudinal sections of banana (*Musa* spp. AAA) roots. DZ, differentiation zone; Ep, epidermis; M, meristem; Pr, protoderm; RC, root cap; RH, root hairs; Xy, xylem. Bars represent 100 μm.

The immunolocalisation with JIM13 antibody recognizing β-D-GlcpA-(1 → 3)-α-D-GalpA-(1 → 2)-α-L-Rha was observed in the root cap, epidermis and root hairs, as well as in the xylem (Fig. 4a–c). The labelling pattern of JIM8, PN16.4B4 and CCRC-M134 antibodies was similar to that of JIM13 antibody. But their antigen levels were much weaker. Nearly no signal was present in the epidermis (Fig. 4d–i).

Negligible or very weak labelling was observed with all the other nine antibodies (JIM12, JIM14, JIM17, JIM101, LM1, MAC204, MAC207, MAC265 and MAC266) recognizing different AG or AGP components in the root cells of the two studied cultivars, both before and after treatments (data not shown). This suggested that above epitopes are depleted and/or not present in the banana roots.

An overview of immunolabelling results from the present study is summarized in Supplementary Table S1.

### The changes in HRGPs in banana roots in response to *Foc* TR4

#### Changes in transcript levels

As shown in Table 1, five extensins and 19 AGPs showed higher expression levels in the susceptible control than in the resistant one. One AGP showed higher level in the susceptible cultivar after wounding (*MaFLA2*-*2*) and other (*MaFLA11*) after pathogen treatment of wounded plants when compared to the resistant cultivar. Only one AGP (*MaAGP23*-*2*) showed higher level in the resistant cultivar after wounding.

Considering extensin transcript level changes, wounding resulted in increased expression of *MaELP*-*2* in the resistant cultivar (Table 2). On the other hand, decrease in *MaPELP*-*3* level in the susceptible cultivar was encountered. Pathogen attack of wounded plants did not result in significant changes in extensin transcript levels in both cultivars. However, the transcript levels of two extensin members were higher in the pathogen inoculated resistant cultivar (inoculation without wounding) than the non-treated control while lower levels of two extensins were observed in the susceptible cultivar. Wounding treatment and pathogen inoculation of wounded plants did not result in changes in AGP expression levels in the susceptible cultivar. However, the transcript levels of four AGPs slightly increased after wounding in the resistant cultivar. Furthermore, two of these four AGP members increased also after pathogen attack of wounded plants (Table 2).

##### qPCR analysis

**Figure 5:**
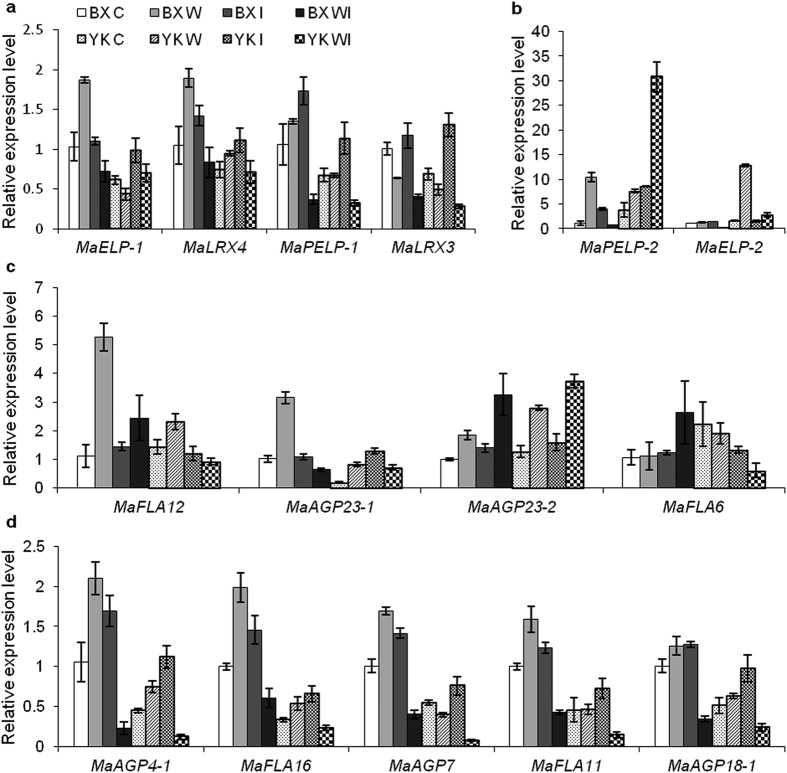
Changes in the transcript levels of extensin (**a**,**b**) and AGP (**c**) members in banana (*Musa* spp. AAA) roots after wounding and infection with *Fusarium oxysporum* f. sp. *cubense*. BX, *Musa* spp. AAA cv. Baxijiao (susceptible); YK, *Musa* spp. AAA cv. Yueyoukang 1 (resistant). AGP, arabinogalactan protein; C, control; W, wounding; I, inoculation of intact plants; WI, inoculation by pathogen after plant wounding. Values are the means ± SE (n = 3). More gene information is available at Supplementary Table S2.

To further validate the results from DGE, six extensin and nine AGP representatives were selected to carry out qPCR. The examination of extensin transcript levels showed that, consistently with DGE, *MaELP*-*1, MaPELP*-*1, MaLRX3* and *MaLRX4* were more expressed in the susceptible control than in the resistant one (Fig. 5a). Among these, the expression of *MaELP*-*1, MaPELP*-*1* and *MaLRX4* increased after wounding but decreased after pathogen inoculation of wounded susceptible cultivar. The expression of these extensins varied after wounding in the resistant cultivar. Increased expressions levels of *MaELP*-*1* were found after pathogen inoculation of wounded plants. Also the transcript levels of *MaLRX3* were down-regulated after both wounding and pathogen attack of wounded plants in both cultivars (Fig. 5a). Transcriptional analysis of other two extensins (*MaPELP*-*2* and *MaELP*-*2*) showed wound-induced overexpression of *MaPELP*-*2* in both cultivars (Fig. 5b), while dramatically increased transcript level of *MaELP*-*2* was observed only in the resistant cultivar after wounding. Moreover, in the resistant cultivar, the transcript level of *MaPELP*-*2* was dramatically up-regulated by pathogen attack of wounded plants. On the other hand, the expression level of *MaPELP*-*2* decreased after pathogen inoculation of wounded susceptible cultivar (Fig. 5b). Pathogen inoculation of intact plants resulted in higher transcription levels of all tested extensins and AGPs except *MaELP*-*2* when compared to the control plants of both cultivars (Fig. 5a,b).

Consistent with the DGE results, AGPs such as *MaAGP4*-*1, MaAGP7, MaAGP18*-*1, MaAGP23-1, MaFLA11* and *MaFLA16* showed higher levels in the susceptible control than in the resistant one (Fig. 5c,d). In most cases, AGPs showed similar changes after treatments. They were up-regulated by wounding followed by a decline after pathogen attack of wounded plants. These included *MaAGP4-1, MaAGP7, MaAGP18*-*1, MaAGP23*-*1, MaFLA12* and *MaFLA16* in both cultivars; *MaAGP7* and *MaFLA11* in the susceptible cultivar. Some other AGPs, however, responded to the treatments differently. The transcript levels of *MaAGP23*-*2* in both cultivars were up-regulated by both wounding and pathogen attack of wounded plants (Fig. 5c). On the other hand, the expression level of *MaAGP7* in the resistant cultivar was down-regulated by both treatments (Fig. 5d), which was not consistent with DGE result (Tables 1 and 2). Typically, pathogen inoculation of intact plants resulted in slight up-regulation of *MaAGP4*-*1, MaAGP7, Ma AGP18*-*1, MaAGP23*-*1, MaAGP23-2, MaFLA11* and *MaFLA16* in both cultivars as well as slight down-regulation of *MaFLA6* in the resistant cultivar (Fig. 5c,d).

#### Changes in protein levels of extensins and AGPs

As described above, only nine out 21 antibodies showed signal in banana roots. Seven (JIM8, JIM11, JIM13, JIM16, JIM20, LM2 and LM14) out of the 9 antibodies with relatively high epitope levels in banana roots were selected to monitor the changes of HRGPs after wounding and pathogen attack using immunofluorescence labelling.

##### Extensins

**Figure 6:**
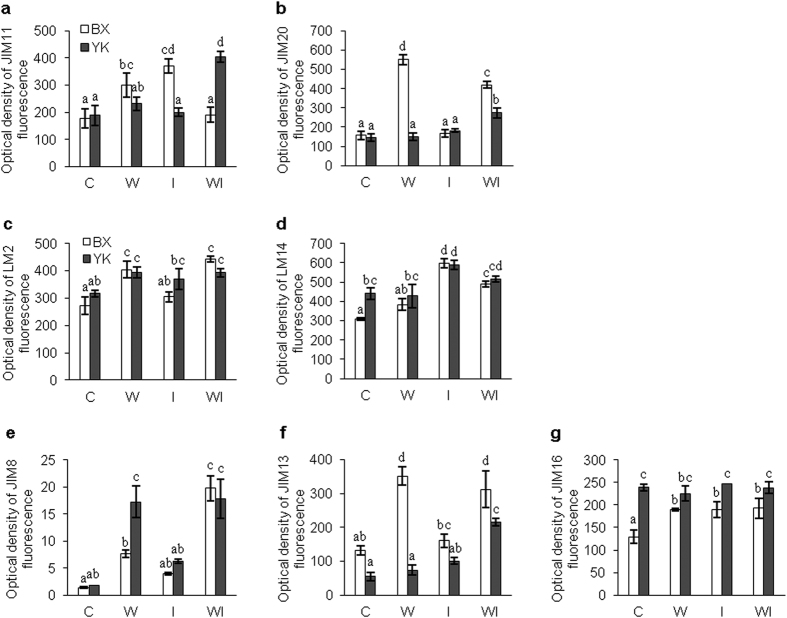
Changes in the protein levels of extensins (**a**,**b**) and AGPs (**c**–**g**) in banana (*Musa* spp. AAA) roots after wounding and infection with *Fusarium oxysporum* f. sp. *cubense*. BX, *Musa* spp. AAA cv. Baxijiao (susceptible); YK, *Musa* spp. AAA cv. Yueyoukang 1 (resistant). AGPs, Arabinogalactan proteins; C, control; W, wounding; I, inoculation of intact plants; WI, inoculation by pathogen after plant wounding. The fluorescence density was calculated by ImageJ software. Values are the means ± SE (n = 3) and different letters indicate significant differences (one-way ANOVA, *P* < 0.05) between treatments.

As shown in Fig. 6a, in the control plants, there was no significant difference in JIM11 antigen level between the resistant and susceptible cultivars. Both wounding and inoculation of intact plants resulted in statistically significant increase of JIM11 fluorescence signal in the susceptible cultivar. Pathogen inoculation of wounded resistant plants also resulted in higher JIM11 fluorescence signal (Fig. 6a). Similarly, the JIM20 signal in control plants was nearly equal in both cultivars (Fig. 6b). Nevertheless, the responses of these two cultivars were different. Wounding resulted in 3.6 fold increase in JIM20 fluorescence in susceptible cultivar, while the signal intensity did not change compared to control after inoculation of intact plants (without wounding). Pathogen inoculation of wounded plants slightly enhanced the JIM20 epitope levels in both cultivars. JIM20 fluorescence signal in resistant cultivar remained unchanged after wounding or pathogen inoculation of intact plants, but it increased 1.5 fold after inoculation of wounded plants (Fig. 6a). These results possibly indicated that extensin labelled by JIM11 positively correlated with the resistance of banana to *Foc* while JIM20 binding extensin positively correlated with the susceptibility to *Foc*.

##### AGPs

As shown in Fig. 6c, there was no significant difference in the abundance of LM2 antigen between the resistant and susceptible control plants. Furthermore, the responses of LM2 binding AGP in both cultivars to wounding and pathogen inoculation of wounded plants were similar. A significant increase in antigen abundance was observed after wounding treatment and pathogen attack of wounded plants in comparison to control. However, when intact plants were inoculated the antigen abundance was not significantly changed (Fig. 6c).

Abundance of LM14 antigen in the resistant cultivar was significantly higher than in the susceptible one in untreated controls. This pattern was not significantly changed by wounding. However, pathogen inoculation of intact plants resulted in a significant increase of LM14 antigen levels in both cultivars as compared to control. Pathogen inoculation after plant wounding showed LM14 antigen levels lower than after inoculation of intact plants, but higher than after wounding without inoculation (Fig. 6d).

We observed low levels of JIM8-recognizing AGPs in both resistant and susceptible cultivars under control conditions (Fig. 6e). Wounding resulted in the significant increase of JIM8 abundance in both cultivars. Resistant cultivar showed higher levels of JIM8 after this treatment. JIM8 epitope levels increased also after inoculation of intact plants, but less in comparison to wounding. Pathogen inoculation after plant wounding resulted in higher JIM8 fluorescence signal especially in the susceptible cultivar, while it remained unchanged in resistant cultivar when compared to wounding alone (Fig. 6e).

AGPs recognized by JIM13 antibody elevated their abundances in response to wounding and combination of inoculation with wounding especially in the susceptible cultivar, while they did not change after inoculation of intact plants (Fig. 6f). The JIM13 fluorescence signal remained weak in the resistant cultivar after wounding and inoculation of intact plants (without wounding), while it increased in response to pathogen inoculation combined with wounding. In all cases, the JIM13 epitope abundance was much higher in the susceptible cultivar than in the resistant one (Fig. 6f). The abundance of JIM16-recognizing AGP was significantly lower in the susceptible control in comparison to the resistant one. Epitope abundances increased almost equally in the susceptible cultivar after wounding treatment and pathogen inoculation of intact plants as well as wounded plants. On the other hand, in the resistant cultivar antigen abundances remained constant, but higher than those in the susceptible cultivar in all cases (Fig. 6g).

Immunolocalization of aforementioned seven antibodies binding extensins or AGPs in banana roots in response to wounding and *Foc* was presented in the Supplementary Figs S1–S4.

In summary, increased sensitivity to *Foc* was followed by higher abundances of majority of AGPs in response to wounding and pathogen inoculation after wounding. On the other hand, we encountered a positive correlation of JIM8 abundance with higher resistance to *Foc* in response to wounding.

## Discussion

As mentioned above, plant cell walls are the first barrier for pathogen penetration to intracellular space in plants. HRGPs, including AGPs and extensins, are highly abundant cell wall components. They contribute to the cell wall architecture and control cell elongation and extension. Moreover, they are also involved in inhibition of the progress of the pathogen during pathogen attack^26^.

In the present study, only two (JIM11 and JIM20) out of five extensin antibodies showed signal in banana roots. These extensins were detected also in banana embryogenic cells, embryos^27^,^28^ and wax gourd (*Benincasa hispida* Cogn)^10^. The subcellular distribution pattern of JIM11 in banana roots is similar as reported in carrot (*Daucus carota L*.)^29^, showing intensive signal in the root cap, epidermis and endodermis, as well as vascular cylinder. Interestingly, the distribution pattern of JIM11 in banana differed from wax gourd, which showed very strong signal in the cortex, endodermis and phloem^10^. Similarly, the JIM20 antibody also showed different labelling pattern in banana and wax gourd. In banana, signal was very strong in the vascular cylinder, moderate in the central root cap but very weak in the cortex. However, the JIM20 epitope level in roots of wax gourd was the highest in the cortex and endodermis, followed by phloem in the vascular cylinder^10^. These further confirmed that extensins are not only differentially distributed in root tissues suggested by Smallwood *et al*.^29^ but also in different plant species^30^.

AGPs are highly glycosylated members of HRGPs and are abundant in all plant organs including roots, as well as in root exudates. They are differentially distributed and developmentally regulated in root tissues^3^. Similarly to extensins, AGPs are differentially distributed in root tissues of different plant species. For example, LM2 recognizing AGP is nearly evenly distributed throughout the cross-section of wax gourd roots except relatively weak signal in the root epidermis^10^. However, in banana, the signal in vascular cylinder was relatively stronger when compared to the other tissues and cells. This AGP was also found in the root epidermis of other plants, such as *Arabidopsis thaliana*^31^ and maize (*Zea mays*)^32^. The LM14 epitope nearly distributed evenly in the elongation zone of banana root, but this was not the case for wax gourd. Difference was also observed in the labelling pattern of JIM16 antibody between banana and wax gourd. Furthermore, different plant species contained different AGP members. For example, MAC204 was very abundant in the cortex and phloem of wax gourd^10^ but not in banana root. The other MAC-series antibody members (MAC207, MAC265 and MAC266) were also detected in roots of many other plant species^33^,^34^,^35^,^36^,^37^,^38^ but they were nearly undetectable in banana roots. In addition, similar set of antibodies were used to investigate the distribution of their epitopes in banana roots, somatic embryos and leaves. The results showed that some epitopes, such as those recognized by JIM11, JIM13, JIM16, JIM20, LM2 and LM14 appeared in both roots and leaves, but some others, such as JIM4 and MAC204 binding AGPs, were differentially expressed in roots or leaves^27^,^28^,^39^. This indicated that AGPs are organ specific.

On the other hand, different plants also showed some similarity. For example, JIM13 binding AGP mainly appeared in the root hairs, epidermis, the xylem and root cap of banana. This epitope was also found in the root cap^38^,^40^,^41^, epidermis^42^ and xylem or phloem cells of *A. thaliana*^40^,^43^. The JIM16 epitopes which appeared the root apical meristem, BLCs, and elongation zone, also was found to be present in the root apical meristem of carrot^33^ and elongating cells of *A. thaliana*^37^.

In recent years, a lot of efforts have been made to reveal the molecular mechanism of banana responses to *Foc* by modern bio-techniques^19^,^20^,^21^,^22^,^23^. However, none of these reports were involved in the responses of banana to wounding, the major factor affecting the infections of this disease^24^. In the present study, we monitored the changes of AGPs and extensins in two banana cultivars after wounding and found that remarkable differences occurred between the resistant and susceptible cultivars. At transcript levels, DGE results indicated that one extensin-like protein and four AGPs were up-regulated by wounding in the resistant cultivar while only one extensin-like protein was down-regulated in the susceptible cultivar. qPCR revealed that 13 and nine out of 14 tested HRGP members in the susceptible and resistant cultivar, respectively, were up-regulated by wounding. At protein levels, the epitope levels of JIM8 and LM2 increased after wounding in both cultivars. Wounding resulted in a significant up-regulation of JIM11, JIM20, JIM13 and JIM16 only in the susceptible cultivar. Similarly, increased AGPs levels were also observed in tomato (*Solanum lycopersicum*)^15^, *Brassica* and *A. thaliana*^44^,^45^ after mechanical wounding. On the contrary, NaAGP4 was found to be rapidly suppressed by tissue wounding^13^. The mRNA of AtAGP31 decreased to about 30% of its original level in response to methyl jasmonate treatment and wounding^14^. These results indicate that different HRGP members possibly played different functions in response of plants to wounding.

In many studies, the up-regulation of extensins or/and AGPs was frequently observed in the hosts. For example, Davies and his co-authors^45^ found that inoculation of *Brassica* petioles with avirulent strains of pathovars of *Xanthomonas campestris* could induce extensins and AGPs recognized by JIM11, JIM20 and MAC204. Similarly, both of JIM16 and LM2 epitopes were up-regulated in both resistant and susceptible cultivars after inoculation of pathogen in wax gourd^10^. Such observations are in agreement with those reports from *A. thaliana* and tomato (*Solanum lycopersicum*). These authors found that overexpression of *A. thaliana* extensin-Ext1 or snakin-2 and extensin-like protein genes limited pathogen invasiveness in the hosts^11^,^46^,^47^. In the present study, the responses of intact and wounded banana plants when exposed to *Foc* were investigated at both of transcription and protein levels. At transcript levels, pathogen attack of intact plants resulted in an increase in nearly all tested extensin and AGP members, except for decreasing *MaFLA6* and *MaFLA12* levels in the resistant cultivar and no obvious change of *MaELP*-*2* in both cultivars. In opposite, only a few extensin and AGP members were up-regulated by pathogen attack of wounded plants. These AGPs or extensins included *MaAGP23*-*2* in both cultivars, *MaELP*-*1* and *MaPELP*-*2* in the resistant cultivar, *MaFLA6* in the susceptible cultivar. In addition, *MaELP*-*2* and *MaPELP*-*2* showed higher transcript level in the resistant cultivar than in the susceptible one, before and after treatments. Furthermore, the RPKM (reads per kilobase of exon model per million mapped reads) value of *MaELP*-*2* was extremely high when compared to the other extensins/AGPs. At protein levels, the responses of intact banana plants to pathogen attack were also different from those of wounded plants. In all cases for both cultivars, pathogen inoculation of intact plants resulted in up-regulation of all 7 tested HRGPs, but solely the increase in levels of LM14 in both cultivars, JIM11 and JIM16 in the susceptible cultivar was significant. For the wounded plants, significant increase was found in abundances of extensins reactive to JIM11and JIM20 antibodies as well as AGP recognized by JIM13 antibody in the resistant cultivar and AGPs reactive to JIM8 and LM14 in the susceptible cultivar after pathogen inoculation. On the contrary, JIM11 and JIM20-binding extensins decreased significantly in the susceptible cultivar. Our results further show that different sets of HGRPs react to *Foc* inoculation in intact plants and wounded banana plants. Their abundance and expression differs also depending on the resistance of the banana plants.

Interestingly, we found that most extensins and AGPs showed higher transcript levels in the susceptible cultivar than in the resistant one, especially in plants after wounding (only excluding *MaELP*-*2, MaPELP*-*2, MaFAL6*, and *MaAGP23-2*). AGPs were found to be essential at the initiation of the dialog, or recognition, between root cells and microbes^38^,^48^ or possibly the pathogen consumes AGP-derived sugars as nutrient source^49^. Similar result was reported by Dobón *et al*.^50^. Four transcription factor mutants from *A. thaliana*, showing enhanced disease susceptibility to necrotrophs, shared a common transcriptional signature of 77 up-regulated genes. Genes encoding secreted Pro/Hyp-rich AGPs, in particular AGP12 and AGP21, were over-expressed in mutants and they contribute to plant disease susceptibility as signalling components. Our results indicate that the above mentioned extensins and AGPs possibly contributed to the susceptibility of banana to the pathogen as signalling components^50^,^51^. On the other hand, some others, such as *MaELP*-*2, MaPELP*-*2* showed higher levels in the controls of resistant plants and they increased only in the resistant cultivar either after wounding (*MaELP*-*2*) or pathogen inoculation of wounded plants (*MaPELP*-*2*). Increase in gene expression in resistant cultivar was found also after pathogen attack of intact plants. These HRGP members possibly contributed to the resistance of banana to the *Foc via* co-operative action between extensin network formation and the electrostatic interaction of additional wall proteins with the extracellular matrix^6^. They may crosslink to each other and form a network which might provide anchorage for lignification and create a barrier impermeable to fungal hyphae^5^. They are also capable to inhibit the germination of pathogen spores and the development of the pathogenic oomycete^51^. These results further confirmed that AGPs and extensins were able to play dual roles in the interactions between plants-hosts interactions. Further research is needed to define precise functions of individual HRGP members and the mechanism underlying.

In the present study, transcript levels *MaELP*-*2, MaPELP*-*2* and the epitope abundance of JIM11 antibody were much more increased in the resistant cultivar after wounding or pathogen inoculation of wounded plants. Other AGPs and extensins (JIM8, JIM13 and JIM20), also showing induction in response to wounding or pathogen attack in resistant cultivar, however in less intensive extent compared to sensitive one, possibly support other defense mechanisms in resistance to Foc.

These results suggested that *MaELP*-*2, MaPELP*-*2* and JIM11 specific extensins might be promising markers of increased banana resistance to *Fusarium* wilt. They might be used for screening of new banana genotypes with increased *Foc* resistance and thus speed up the breeding programs on banana resistance to *Fusarium* wilt. Many efforts have been made for this goal so far. The most widely employed bioassay is a pot system^52^, followed by a hydroponic system^53^. Previously, Wu *et al*.^54^ developed an *in vitro* screening system. When compared to these systems, our protocol is beneficial due to his lower time requirements^54^. However, for implementation to breeding programs, further validation with a large quantity of germplasm with different resistance to *Foc* has to be carried out.

## Material and Methods

### Materials

Two banana cultivars, *Musa* spp. AAA cv. ‘Baxijiao’ and ‘Yueyoukang 1’ were used in this study. ‘Yueyoukang 1’, a cultivar from Cavendish selection GCTCV-218, is highly resistant to *Fusarium oxysporum* f.sp. *cubense* tropical race 4 (*Foc* TR4) while ‘Baxijiao’ is highly susceptible to this pathogen^55^.

*Foc* TR4 was used in the present study for banana inoculation with pathogen. This pathogen was obtained from Lab. of Fungus, South China Agricultural University.

### Inoculation of banana cultivars with pathogen

Preparation of pathogen and the inoculation of two banana cultivars with this *Foc* TR4 were carried out according to the protocol described in our previous work^25^. In brief, two weeks after induction of roots from tissue cultured banana plants, one root of each plant was cut off to facilitate the penetration of the pathogen. Afterwards, such treated plants were transferred to medium containing *Foc* TR4 at final concentration of 5 × 10^2^ conidia per ml (inoculation treatment after wounding). Plants transferred to a medium without fungus served as the cut controls (wounding treatment). Intact plants transferred to medium containing *Foc* TR4 served as inoculation treatment. Intact plants cultured in a medium without fungus served as the non-cut control (the control of wounding treatment). Three replicates (each with 6 plants in a bottle) were set for each treatment. The samples were collected 24 h after treatments. RNA extracted from the roots was subjected to DGE and qPCR analysis. Values reported represent the average of three or two biological replicates for qPCR and DGE, respectively.

### Digital gene expression

#### RNA preparation

The total RNA was extracted from the root tips (about 1.5 cm) of banana cv. ‘Baxijiao’ and ‘Yueyoukang 1’ using the QIAGEN RNeasy plant mini kit (QIAGEN, Valencia, CA), both before and after pathogen inoculation, and treated with RNase-free DNase I (Promega, Madison, Wisconsin, USA). The degradation and contamination of the RNA was monitored with 1% agarose gels while the purity was check using the NanoPhotometer^®^ spectrophotometer (IMPLEN, CA, USA). The concentration of the total RNA was measured by Qubit^®^ RNA Assay Kit in Qubit^®^ 2.0 Flurometer (Life Technologies, CA, USA). RNA integrity was assessed using the RNA Nano 6000 Assay Kit of the Bioanalyzer 2100 system (Agilent Technologies, CA, USA).

#### Library preparation for DGE sequencing

A total amount of 3 μg RNA per sample was used as input material for the RNA sample preparations. Sequencing libraries were generated using NEBNext^®^ Ultra™ RNA Library Prep Kit for Illumina^®^ (NEB, USA) according to manufacturer’s recommendations. Index codes were added to attribute sequences to each sample. mRNA was purified from total RNA using poly-T oligo-attached magnetic beads. Fragmentation was carried out using divalent cations under elevated temperature in NEBNext First Strand Synthesis Reaction Buffer (5X). The first strand cDNA was synthesized using random hexamer primer and M-MuLV Reverse Transcriptase (RNase H-) while the second one was done using DNA polymerase I and RNase H. Remaining overhangs were converted into blunt ends *via* exonuclease/polymerase activities. After adenylation of 3′ ends of DNA fragments, NEBNext Adaptor with hairpin loop structure was ligated to prepare for hybridisation. In order to select cDNA fragments of preferentially 150~200 bp in length, the library fragments were purified with AMPure XP system (Beckman Coulter, Beverly, USA). 3 μl USER Enzyme (NEB, USA) was used with size-selected, adaptor-ligated cDNA at 37 °C for 15 min followed by 5 min at 95 °C before PCR. PCR was performed with Phusion High-Fidelity DNA polymerase, Universal PCR primers and Index (X) Primer. Finally, PCR products were purified (AMPure XP system) and library quality was assessed on the Agilent Bioanalyzer 2100 system.

The clustering of the index-coded samples was performed on a cBot Cluster Generation System using TruSeq PE Cluster Kit v3-cBot-HS (Illumina) according to the manufacturer’s instructions. After cluster generation, the library preparations were sequenced on an Illumina Hiseq 2000/2500 platform and 100 bp/50 bp single-end reads were generated.

#### Data Analysis

##### Quality control

Firstly, raw reads of fastq format were processed through in-house perl scripts. The clean reads were obtained after removal of reads containing adapter, ploy-N and at low quality from raw data. All the downstream analyses were based on the clean data.

##### Reads mapping to the reference genome

Reference genome and gene model annotation files were downloaded from NCBI database directly. Index of the reference genome was built using Bowtie v2.0.6 and single-end clean reads were aligned to the reference genome using TopHat v2.0.9. TopHat was selected as the mapping tool. Bowtie v0.12.9 was used to aligned single-end clean reads to the unigene sequences.

##### Quantification of gene expression level

HTSeq v0. 6.1 was used to count the reads numbers mapped to each gene. The RPKM of each gene was calculated based on the length of the gene and reads count mapped to this gene. RSEM was employed to count the reads numbers mapped to each unigene.

##### Differential expression analysis

Differential expression analysis of two conditions/groups (two biological replicates per condition) was performed using the DESeq R package (1.10.1). The *P*-values were adjusted using the Benjamini and Hochberg’s approach for controlling the false discovery rate. Genes with an adjusted *P*-value < 0.05 found by DESeq were assigned as differentially expressed.

### qPCR detection of the transcript levels of representative AGP and extensin members

The total RNA was extracted as described previously. The protocol for qPCR analysis was performed as described by Ma *et al*.^25^. The primers for qPCR are listed in the Supplementary Table S3.

### Immuno-fluorescence labelling

The fixation and immuno-labelling methods were performed according to our previous work^28^. AGP and extensin recognizing antibodies used for immunofluorescence labelling were the same as in Yan *et al*.^39^ and Xie *et al*.^10^ respectively. For quantification of fluorescence signal, the integrated density was measured with Image J 1.44 software (n = 3 sections of roots, in three biological replicates).

## Additional Information

**How to cite this article:** Wu, Y. *et al*. Expression and distribution of extensins and AGPs in susceptible and resistant banana cultivars in response to wounding and *Fusarium oxysporum. Sci. Rep.* **7**, 42400; doi: 10.1038/srep42400 (2017).

**Publisher's note:** Springer Nature remains neutral with regard to jurisdictional claims in published maps and institutional affiliations.

## Supplementary Material

Supplementary Information

## Figures and Tables

**Table 1:** Differences in digital gene expression levels of extensins and AGPs between two banana (*Musa* spp. AAA) cultivars before and after wounding and infection with *Fusarium oxysporum* f. sp. *cubense.*

Gene family	Gene name	log_2_FoldChange		
		YK-C *vs* BX-C	YK-W *vs* BX-W	YK-WI *vs* BX-WI
Extensins	*MaPELP*-*3*	3.63		
	*MaPELP*-*1*	1.21		
	*MaLRX3*	1.20		
	*MaLRX4*	1.02		
	*MaELP*-*1*	1.02		
AGPs	*MaAGP23*-*1*	2.19		
	*MaAGP20*	1.73		
	*MaAGP19*-*1*	1.55		
	*MaFLA12*	1.48		
	*MaFLA7*	1.39		
	*MaAGP7*	1.38		
	*MaAGP16*	1.38		
	*MaFLA6*	1.32		
	*MaFLA2*-*1*	1.28		
	*MaFLA16*	1.28		
	*MaFLA8*	1.21		
	*MaAGP18*-*2*	1.16		
	*MaAGP26*	1.14		
	*MaAGP4*-*2*	1.13		
	*MaAGP4*-*1*	1.10		
	*MaAGP18*-*1*	1.06		
	*MaFLA1*	1.03		
	*MaFLA13*	1.03		
	*MaFLA11*	1.18		1.44
	*MaFLA2*-*2*		1.03	
	*MaAGP23*-*2*		−1.09	

BX, *Musa* spp. AAA cv. Baxijiao (susceptible); YK, *Musa* spp. AAA cv. Yueyoukang 1 (resistant). AGP, arabinogalactan protein; C, control; W, wounding; WI, inoculation by pathogen after plant wounding. Values are the means (n = 2) and *P*-value < 0.05. More gene information is available at Supplementary Table S2.

**Table 2:** The responses of extensins and AGPs in banana (*Musa* spp. AAA) to wounding and *Fusarium oxysporum* f. sp. *cubense* as revealed by digital gene expression analysis.

Gene family	Gene name	log_2_FoldChange		
		YK-C *vs* YK-W	YK-W *vs* YK-WI	YK-C *vs* YK-WI
Extensins	*MaELP*-*2*	1.79		1.83
	*MaPELP*-*2*			4.80
AGPs	*MaAGP4*-*1*	1.12		1.32
	*MaAGP7*	1.06		1.31
	*MaAGP18*-*1*	1.01		
	*MaAGP19*-*2*	1.01		
	*MaASD1*	−1.59		−1.81
**Gene family**	**Gene name**	**log**_**2**_**FoldChange**		
		**BX-C** ***vs*** **BX-W**	**BX-W** ***vs*** **BX-WI**	**BX-C** ***vs*** **BX-WI**
Extensins	*MaPELP*-*3*	−1.36		−2.06
	*MaLRX3*			−1.40

BX, *Musa* spp. AAA cv. Baxijiao (susceptible); YK, *Musa* spp. AAA cv. Yueyoukang 1 (resistant). AGP, arabinogalactan protein; C, control; W, wounding; WI, inoculation by pathogen after wounding. Values are the means (n = 2) and *P*-value < 0.05. More gene information is available at Supplementary Table S2.
