# Supplementary material for: Expression and distribution of extensins and AGPs in susceptible and resistant banana cultivars in response to wounding and Fusarium oxysporum
Source: Sci Rep. 2017 Feb 20;7:42400. doi: 10.1038/srep42400 (PMC5316987; doi:10.1038/srep42400)
Supplement: Supplementary Information [file srep42400-s1.pdf]

# Expression and distribution of extensins and AGPs in susceptible and resistant banana cultivars in response to wounding and *Fusarium oxysporum*.

Yunli Wu[1], Wei Fan[1], Xiaoquan Li[2], Houbin Chen[1], Tomáš Takáč[3], Olga Šamajová[3], Musana Rwalinda Fabrice[1], Ling Xie[1], Juan Ma[1], Jozef Šamaj[3], Chunxiang Xu[1]*

*Author for correspondence: Chunxiang Xu
Email: chxxu@scau.edu.cn

## Supplementary Figures S1–S4 and Tables S1–S3

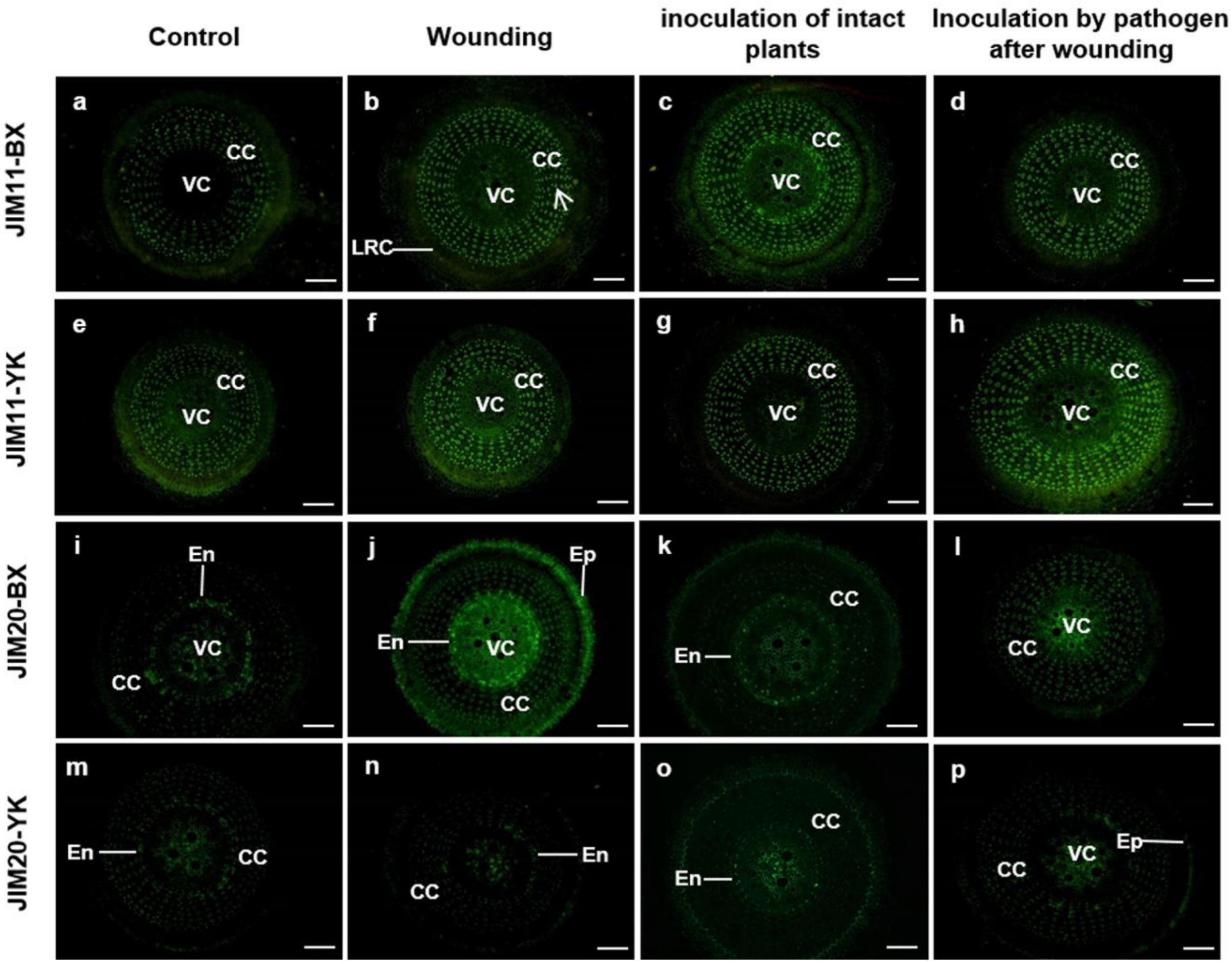


**Figure S1. Immunolocalization of JIM11 and JIM20 binding extensins in banana (*Musa* spp. AAA) roots in response to wounding and *Fusarium oxysporum* f. sp. *cubense*.** In all cases, cross sections through roots are presented. BX, Baxijiao (susceptible); YK, Yueyoukang 1 (resistant). CC, cortical cells; En, endodermis; Ep, epidermis; LRC, lateral root cap; VC, vascular cylinder. Arrow points to tricellular cell–cell junction. Bars represent 100 µm.

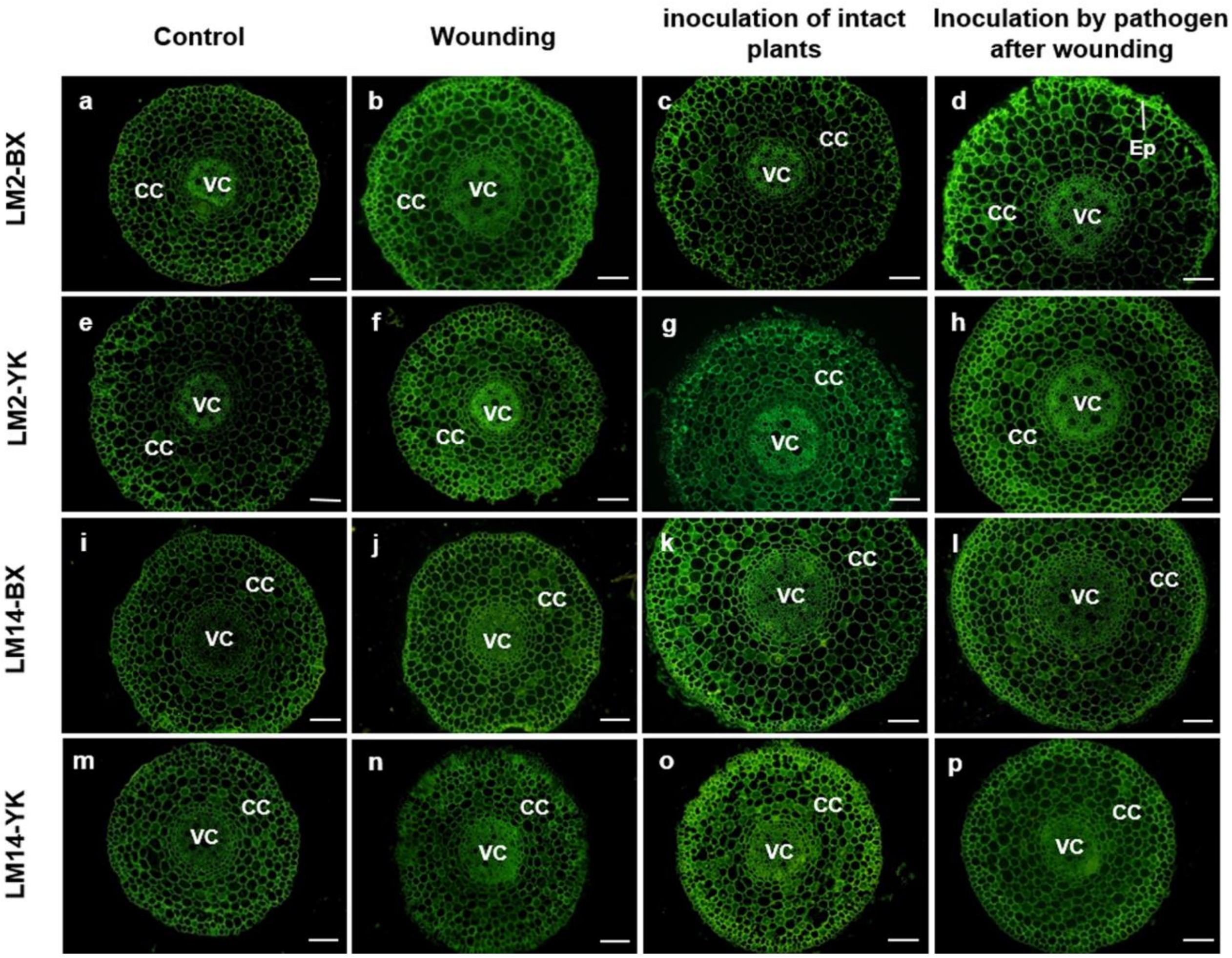


**Figure S2. Immunolocalization of LM2 and LM14 binding AGPs in banana (*Musa* spp. AAA) roots in response to wounding and *Fusarium oxysporum* f. sp. *cubense*.** In all cases, cross sections through roots are presented. BX, Baxijiao (susceptible); YK, Yueyoukang 1 (resistant). CC, cortical cells; Ep, epidermis; VC, vascular cylinder. Bars represent 100 µm.

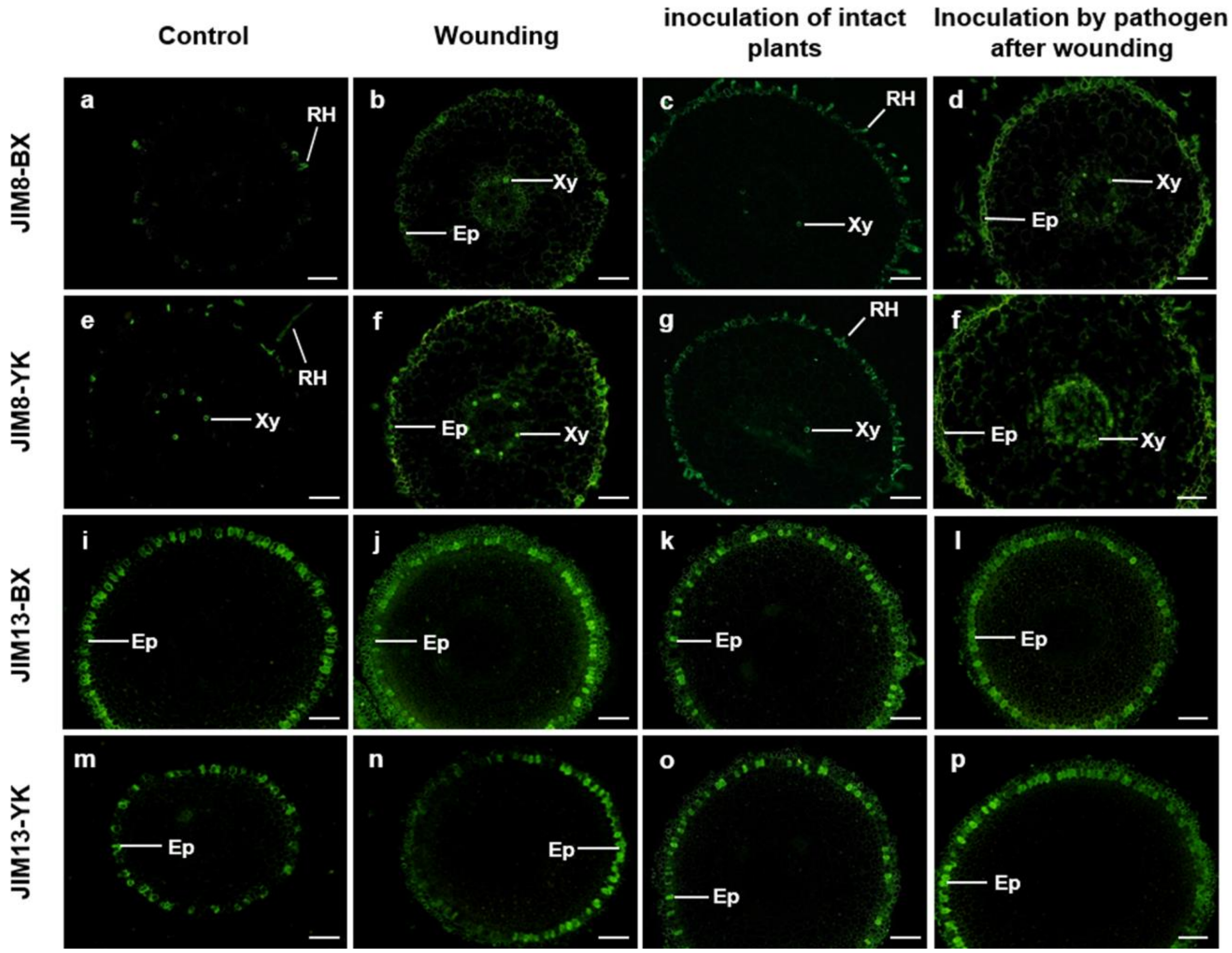


**Figure S3. Immunolocalization of JIM8 and JIM13 binding AGPs in banana (*Musa* spp. AAA) roots in response to wounding and *Fusarium oxysporum* f. sp. *cubense*.** In all cases, cross sections through roots are presented. BX, Baxijiao (susceptible); YK, Yueyoukang 1 (resistant). Ep, epidermis; RH, root hairs; Xy, xylem. Bars represent 100 μm.

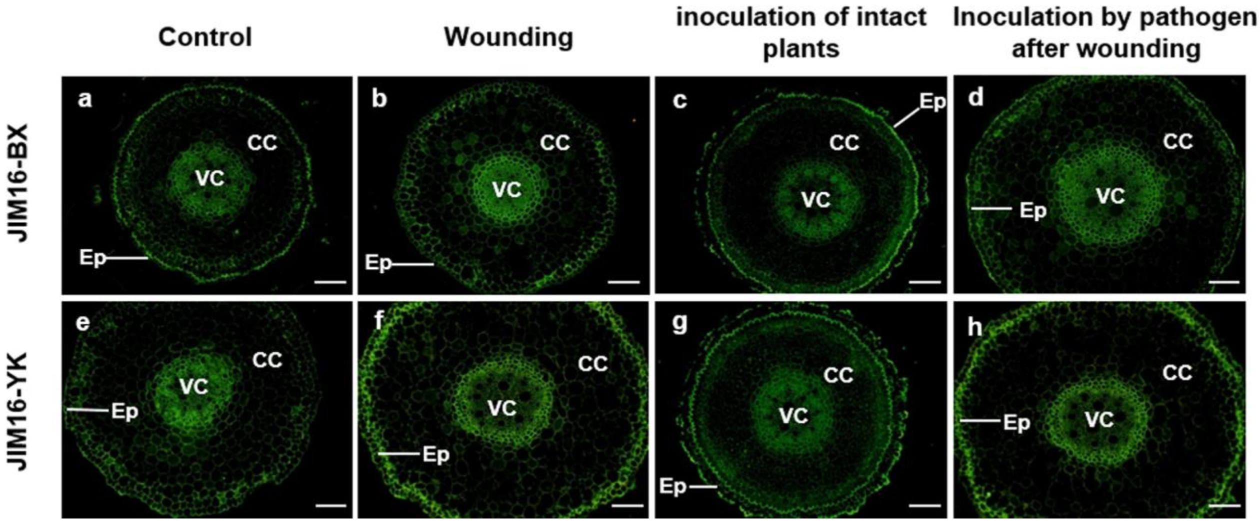


**Figure S4. Immunolocalization of JIM16 binding AGPs in banana (*Musa* spp. AAA) roots in response to wounding and *Fusarium oxysporum* f. sp. *cubense*.** In all cases, cross sections through roots are presented. BX, Baxijiao (susceptible); YK, Yueyoukang 1 (resistant). CC, cortical cells; Ep, epidermis; VC, vascular cylinder. Bars represent 100 μm.

**Table S1. The intensity of immunofluorescence labelling of banana (*Musa* spp. AAA) roots with different extensins and arabinogalactan proteins antibodies.**

| Antibody used | Root cap | Epidermis | Cortical cells | Endodermis | Root hairs | BLCs | Vascular cylinder | | |
|---|---|---|---|---|---|---|---|---|---|
| | | | | | | | Pericycle | Phloem | Xylem and VP |
| JIM11 | ++ | + | + | ++ | - | - | ± | ± | ++ |
| JIM20 | ++ | ± | ± | + | - | - | + | + | ++++ |
| LM2 | ± | ± | +++ | + | - | - | + | ++++ | ++++ |
| LM14 | + | ± | ++ | ++ | - | - | ++ | ++ | ++ |
| JIM16 | ++++ | ++++ | ± | +++ | - | ++++ | +++ | ++++ | + |
| JIM13 | ++++ | ++++ | - | - | ++++ | ++++ | - | - | ++++ |
| JIM8 | +++ | ± | - | - | +++ | - | - | - | ++++ |
| PN16.4B4 | ++ | ± | - | - | ++ | - | - | - | ++ |
| CCRC-M134 | + | ± | - | - | ± | - | - | - | ± |

Increasing intensity of immunolabelling was evaluated as: – (no labelling), ± (very weak), + (weak), ++ (middle), +++ (strong), ++++ (very strong). BLCs, border-like cells, VP, vascular parenchyma.

**Table S2. Gene information.**

| Gene famliy | Gene ID | Gene name | Gene description |
|---|---|---|---|
| Extensins | LOC103994065 | *MaELP-1* | extensin-like protein |
| | LOC103997407 | *MaELP-2* | extensin-like protein |
| | LOC103989053 | *MaLRX3* | pollen-specific leucine-rich repeat extensin-like protein 3 |
| | LOC103997909 | *MaLRX4* | leucine-rich repeat extensin-like protein 4 |
| | LOC103973738 | *MaPELP-1* | pistil-specific extensin-like protein |
| | LOC103991051 | *MaPELP-2* | pistil-specific extensin-like protein |
| | LOC103996093 | *MaPELP-3* | pistil-specific extensin-like protein |
| AGPs | LOC103975630 | *MaAGP4-1* | classical arabinogalactan protein 4-like |
| | LOC103982666 | *MaAGP4-2* | classical arabinogalactan protein 4-like |
| | LOC103973630 | *MaAGP7* | classical arabinogalactan protein 7-like |
| | LOC104000828 | *MaAGP16* | arabinogalactan peptide 16-like |
| | LOC103979425 | *MaAGP18-1* | lysine-rich arabinogalactan protein 18-like |
| | LOC103992896 | *MaAGP18-2* | lysine-rich arabinogalactan protein 18-like |
| | LOC103975936 | *MaAGP19-1* | lysine-rich arabinogalactan protein 19-like |
| | LOC103978045 | *MaAGP19-2* | lysine-rich arabinogalactan protein 19-like |
| | LOC103991644 | *MaAGP20* | arabinogalactan peptide 20-like |
| | LOC103985508 | *MaAGP23-1* | arabinogalactan peptide 23-like |
| | LOC103997732 | *MaAGP23-2* | arabinogalactan peptide 23-like |
| | LOC103993392 | *MaAGP26* | classical arabinogalactan protein 26-like |
| | LOC103995161 | *MaASD1* | alpha-L-arabinofuranosidase 1-like |
| | LOC103968476 | *MaFLA1* | fasciclin-like arabinogalactan protein 1 |
| | LOC103972757 | *MaFLA2-1* | fasciclin-like arabinogalactan protein 2 |
| | LOC103998373 | *MaFLA2-2* | fasciclin-like arabinogalactan protein 2 |
| | LOC103987751 | *MaFLA6* | fasciclin-like arabinogalactan protein 6 |
| | LOC103980339 | *MaFLA7* | fasciclin-like arabinogalactan protein 7 |
| | LOC103970284 | *MaFLA8* | fasciclin-like arabinogalactan protein 8 |
| | LOC103986177 | *MaFLA11* | fasciclin-like arabinogalactan protein 11 |
| | LOC103988759 | *MaFLA12* | fasciclin-like arabinogalactan protein 12 |
| | LOC104001024 | *MaFLA13* | fasciclin-like arabinogalactan protein 13 |
| | LOC103983519 | *MaFLA16* | fasciclin-like arabinogalactan protein 16 |

AGPs, arabinogalactan proteins

**Table S3. Primers for qPCR.**

| Gene family | Gene name | Primer sequences (Forward/ Reverse) | Production size (bp) |
|---|---|---|---|
| | *18S rRNA* | CCTGAGAAACGGCTACCACAT<br>CACCAGACTTGCCCTCCA | 171 |
| Extensins | *MaELP-1* | TTCCCACCATCATCTCCAAG<br>GGTCTTGTTGAGCTGCCTCTA | 119 |
| | *MaELP-2* | TACAAGACCATCCCTGAGCAC<br>ACACTGCGTCATCTTCAACG | 122 |
| | *MaLRX3* | GTGTCGTTTGTAGCGATCAGG<br>CATGGAGAGAGCGGTTTACAG | 134 |
| | *MaLRX4* | TGTAGGCGTGTCCTATGCTTC<br>AAAGGAAGAGATAGGGGGAGTG | 100 |
| | *MaPELP-1* | CTCTCTACACCGCAGGCTTCT<br>CCTCTCATCAAACGACTACCG | 117 |
| | *MaPELP-2* | GTCCACTGTACTCGAGCTTGC<br>GCTCAAAGGCTCAACACAGAG | 132 |
| AGPs | *MaAGP4-1* | CCGTTGCCATTTTGGTCA<br>GTCCATCACAACGGGGATACT | 130 |
| | *MaAGP7* | CCTTACCTTCAGCTGGGTCA<br>AAACCGTGCGATCAGTATCC | 121 |
| | *MaAGP18-1* | GCCGACCAGACAAGTGGT<br>ATGTAGTGTCTCGGGGAGCTT | 116 |
| | *MaAGP23-1* | GCTCTCCTTCTTCGCCTTCTA<br>CTGGTGGAGCTGACAACAATC | 104 |
| | *MaAGP23-2* | GACGGCGAGAGAGTCTGG<br>GCCTGCAAAGGAGAAGAAGA | 106 |
| | *MaFLA6* | CAAGCAAGCCAAAGAGGAAG<br>AGAGAAGGTGACTGCCCAAC | 106 |
| | *MaFLA11* | GCCAAGAAGCAGAAACCAAG<br>CACTCAGAAGCTCCACCACA | 159 |
| | *MaFLA12* | CGGGAATTTCAACTCCTTCA<br>GGTCACGTTGAGCTTGAACA | 292 |
| | *MaFLA16* | GAAGTTGGGTGCAGTTTGCT<br>GGCAGGTGGTAACATTTGGT | 130 |

AGPs, arabinogalactan proteins
